# A Novel Niosome-Encapsulated Essential Oil Formulation to Prevent *Aspergillus flavus* Growth and Aflatoxin Contamination of Maize Grains During Storage

**DOI:** 10.3390/toxins11110646

**Published:** 2019-11-06

**Authors:** Marta García-Díaz, Belén Patiño, Covadonga Vázquez, Jessica Gil-Serna

**Affiliations:** Department of Genetics, Physiology and Microbiology, Faculty of Biology, University Complutense of Madrid, Jose Antonio Novais 12, 28040 Madrid, Spain; martga43@ucm.es (M.G.-D.); belenp@ucm.es (B.P.); covi@ucm.es (C.V.)

**Keywords:** essential oils, *Satureja montana*, *Origanum virens*, *Aspergillus flavus*, aflatoxin, corn, nanoparticles

## Abstract

Aflatoxin (AF) contamination of maize is a major concern for food safety. The use of chemical fungicides is controversial, and it is necessary to develop new effective methods to control *Aspergillus flavus* growth and, therefore, to avoid the presence of AFs in grains. In this work, we tested in vitro the effect of six essential oils (EOs) extracted from aromatic plants. We selected those from *Satureja montana* and *Origanum virens* because they show high levels of antifungal and antitoxigenic activity at low concentrations against *A. flavus*. EOs are highly volatile compounds and we have developed a new niosome-based encapsulation method to extend their shelf life and activity. These new formulations have been successfully applied to reduce fungal growth and AF accumulation in maize grains in a small-scale test, as well as placing the maize into polypropylene woven bags to simulate common storage conditions. In this latter case, the antifungal properties lasted up to 75 days after the first application.

## 1. Introduction

Aflatoxins (AFs) are secondary metabolites produced primarily by *Aspergillus flavus* and *Aspergillus parasiticus*. Aflatoxin B_1_, B_2_, G_1_, and G_2_ (AFB_1_, AFB_2_, AFG_1_, and AFG_2_) are the most important ones, with AFB_1_ being the most toxic naturally occurring human carcinogen [1,2]. The International Agency for Research on Cancer (IARC) has classified the “naturally occurring mixes of aflatoxins” as Group 1 carcinogens in humans [3].

AFs contaminate a variety of staple crops including cereals (maize, sorghum, barley, oat, rye, rice, and wheat), soya, dry nuts (nuts, pistachios, almonds, and hazelnuts), cottonseed, coffee, cacao, and spices [4].

According to the Food and Agriculture Organization of the United Nations (FAO) [5], maize is one of the most important cereals, with an annual worldwide production of 1134 million tons in 2017, and most of it is intended for direct human and animal consumption. Moreover, maize and its derivatives are considered the main source of AFs worldwide [6]. For all these reasons, the European Union established strict regulations regarding maximum permitted AF levels for maize [7].

The impact of AF contamination of agri-food products is significant. It causes important economic losses because infected products cannot be sold and the contamination also raises veterinary and health costs. Establishing adequate controls to avoid AFs in the food chain is thus essential [8].

Different strategies to prevent AF contamination have been proposed to reduce fungal development in the field or during storage. Applying good agricultural practices and maintaining adequate humidity and temperature in silos are indispensable in reducing fungal growth and, therefore, mycotoxin contamination [9]. Chemical compounds are useful in preventing fungal growth and, for a long time, have been widely used both in the field and during storage to prevent mycotoxin contamination [10]. However, synthetic fungicides are in the spotlight and consumers are now demanding safer foodstuffs that are produced using sustainable and ecofriendly methods. The indiscriminate use of chemical fungicides has important drawbacks, including residue on grain that threatens human and animal health or causes extensive environmental contamination [11]. Moreover, the indiscriminate use of fungicides has caused an increased number of resistant isolates, which makes it very difficult to effectively control fungal growth [12].

The risks of using synthetic chemicals have increased public awareness and demand for safer and ecofriendly products and, in this context, natural plant extracts are now considered good alternatives. Essential oils (EOs) extracted from aromatic plants have demonstrated strong antibacterial, antifungal, and food preservative properties, together with low toxicity, fewer environmental effects, and wider public acceptance [13]. Many EO-based formulations are listed on the generally recognized as safe (GRAS) list, fully approved by the Food and Drug Administration, and are currently commercially available as food preservatives and/or agricultural supplements [14]. Several EOs have been reported to not only reduce growth in toxigenic fungal species, but also to interfere to some extent in mycotoxin biosynthesis. Da Silva et al. [15] reported that *Rosmarinus officinalis* EO has a strong effect against *Fusarium verticillioides,* as it showed the ability to rupture the cell wall and inhibit the production of fumonisins. *Aspergillus flavus* growth and its ability to produce AFs were also significantly affected by treatment with *Origanum virens* and *Ageratum conyzoides* EOs in corn and soybeans [16], and using *Mentha spicata* EO in chickpeas [17].

Therefore, the use of EOs to prevent fungal growth during maize storage could be a sustainable solution to minimize food losses owing to mycotoxin contamination. However, their direct application in food products seems to be limited because of their high volatility, low water solubility, and susceptibility to oxidation [18]. To solve these problems, various encapsulation techniques have been developed that can preserve EOs through a physical or chemical interaction with a matrix that maintains the compounds for a longer time [19]. Encapsulation of EOs also increases stability against oxidation, which helps to prolong their antimicrobial activity [18,20]. Different methods of encapsulation have also been demonstrated to enhance the antifungal and antiaflatoxigenic properties when applied to control *A. flavus* [21].

These encapsulation particles form a protective film that isolates the nucleus that contains the active agent. The composition of the particles should be carefully chosen depending on the encapsulated compound. To date, several natural and synthetic matrices have been successfully used to encapsulate EOs including polyethylene, carbohydrates, proteins, lipids, and gum [22]. The choice of the encapsulation material is a crucial step in developing an appropriate application method for EOs. Different parameters should be taken into account such as the polarity, solubility, and volatility of the active compounds, as well as the composition of the food matrix [23]. Niosomes are lipid-based systems, composed by non-toxic self-assembly vesicles, with a single or multiple layered structure, which are able to encapsulate hydrophobic and hydrophilic compounds [24]. Niosomes are biodegradable, easily stored and handled, and present low toxicity, which are important advantages for their application in the food industry [24].

The aim of this work was to evaluate the in vitro antifungal and antitoxigenic effects of different aromatic plant EOs and to design an effective niosome-based encapsulation protocol to avoid AF contamination during maize storage.

## 2. Results

### 2.1. The Efficacy of Plant Essential Oils Against Fungal Growth and Mycotoxin Production

**Figure 1:**
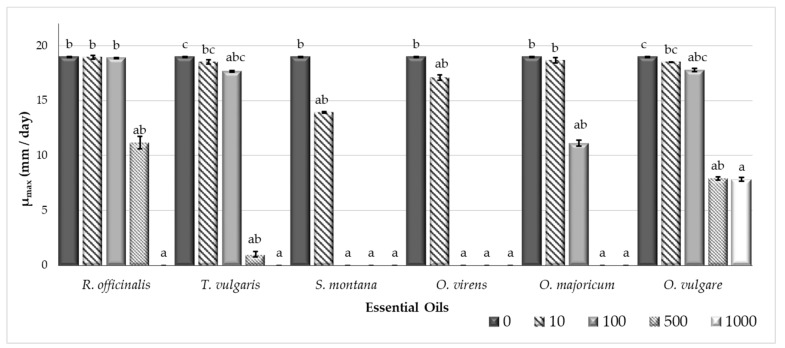
*Aspergillus flavus* S.44-1 growth rate (mm/day) at different concentrations (0, 10, 100, 500, and 1000 µg/mL) of essential oils (*R. officinalis, T. vulgaris, S. montana, O. virens, O. majoricum*, and *O. vulgare*). Each value is the mean of three replications and the thin vertical bars represent the standard error of the corresponding data. Groups with the same letter are not significantly different (*p* > 0.05).

**Figure 2:**
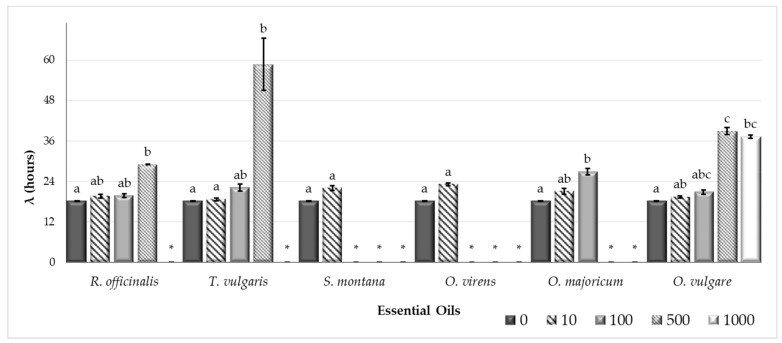
*Aspergillus flavus* S.44-1 lag phase (h) at different concentrations (0, 10, 100, 500, and 1,000 µg/mL) of essential oils (*R. officinalis, T. vulgaris, S. montana, O. virens, O. majoricum,* and *O. vulgare*). Each value is the mean of three replications and the thin vertical bars represent the standard error of the corresponding data. Groups with the same letter are not significantly different (*p* > 0.05). * No data.

Figure 1 and Figure 2 show the results for the *Aspergillus flavus* growth rate (µ, mm/day) and the lag phase prior to growth (λ, h), respectively, in CYA (Czapek Yeast Autolysate Agar) plates supplemented with different essential oils (EOs) (*Rosmarinus officinalis*, *Thymus vulgaris*, *Satureja montana*, *Origanum virens*, *O. majoricum*, and *O. vulgare*) at several different concentrations (0, 10, 100, 500, and 1000 µg/mL).

All EOs tested had a significant effect on the *A. flavus* growth rate at the maximum concentration (Figure 1). Fungal growth was completely inhibited, except for the *O. vulgare* EO. For the EOs of *O. virens* and *S. montana*, total inhibition was reached at 100 µg/mL. *Thymus vulgaris* and *O. majoricum* EOs also showed reductions of at least 95% at 500 µg/mL.

The lag phase got longer as the EO concentration increased (Figure 2). It was not possible to calculate the lag phase when the EO treatment completely inhibited growth in the plates.

Aflatoxin production (AFB_1_, AFB_2_, AFG_1_ y AFG_2_) was significantly reduced compared with the control group in all treatments at the highest concentration tested (1000 µg/mL). No AFs were detected at these concentrations (Table 1) in any case, except for the *O. vulgare* EO treatment, which achieved reductions of more than 97% in all toxins.

AFB_1_ production was significantly affected at 500 µg/mL, with reductions of nearly 100% in all of the EOs tested. The same results were obtained when *O. virens* and *S. montana* EOs were applied at 100 µg/mL.

AFB_2_ production was also significantly reduced by least 90% compared with the control plates after treatment with *R. officinalis, T. vulgaris, O. vulgare*, and *O. majoricum* EOs at 500 µg/mL. The most effective treatments, reaching complete inhibition of AFB_2_ production at 100 µg/mL, were *O. virens* and *S. montana* EOs.

Aflatoxin G_1_ was not detected on *S. montana* and *O. virens* at 100 µg/mL. The rest of the EOs showed a reduction greater than 80% at 500 µg/mL. In all cases, AFG_2_ concentration was below the detection limit (<0.0025 µg/g agar), except in the case of control plates without treatment.

### 2.2. Techniques for the Application of Essential Oils to Prevent Fungal Growth and Mycotoxin Production

The main nanocapsule characteristics of the *O. virens* and *S. montana* niosomes can be found in Table 2. According to these data, both encapsulation processes yield high quality niosomes with low aggregation of nanoparticles. The particle size of both samples was approximately 140 nm with a polydipersity index (PDI) of 0.251 and a ζ-potential of –14 mV.

#### 2.2.1. Small-Scale Assay

**Figure 3:**
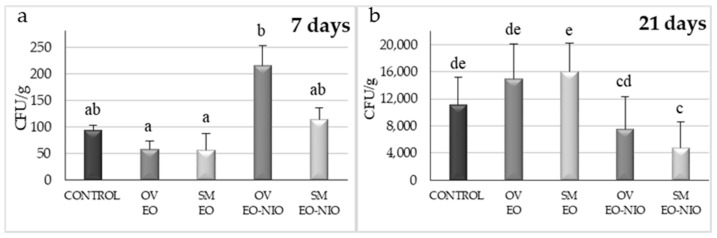
Effect of *S. montana* (SM) and *O. virens* (OV) by direct contact (essential oil, EO) and immobilized in niosomes (EO-NIO) on corn grains inoculated with *A. flavus*, incubated for 7 (**a**) and 21 days (**b**). Each values is the mean of three replications and the thin vertical bars represent the standard error of the corresponding data. Groups with the same letter are not significantly different (*p* > 0.05). CFU, colony forming units.

*Satureja montana* and *O. virens* EOs applied by direct contact to artificially inoculated maize grains and incubated at 28 °C reduced *A. flavus* growth compared with the control group without EOs after seven days of incubation, although no significant differences were observed (Figure 3a). When these EOs were applied in their niosome-encapsulated form and incubated for seven days, colony forming units (CFU) per gram values were slightly increased, although no significant differences were found. When incubation time was extended to 21 days, the opposite effect was observed (Figure 3b). EOs applied by direct contact seemed to lose their effect over time. However, when these compounds were encapsulated in niosomes, *S. montana* EO reduced fungal growth by 58% and, in the case of *O. virens*, this reduction was 32% with respect to the control group.

**Figure 4:**
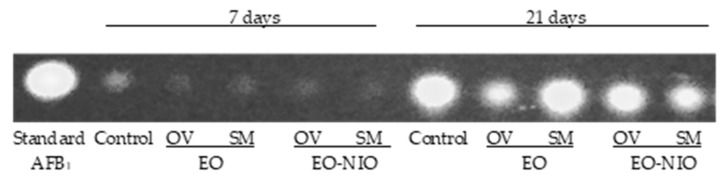
Effect of *S. montana* (SM) and *O. virens* (OV) by direct contact (EO) and encapsulated in niosomes (EO-NIO) on aflatoxin (AF) B_1_ concentration of corn grains inoculated with *A. flavus*, incubated for 7 and 21 days. The standard corresponds to the application of purified AFB_1_ (0.05 mg/mL).

AFB_1_ production was low at the short incubation time (7 days) and very significant for longer periods (21 days) (Figure 4). In the thin layer chromatography (TLC) analysis, the intensity and thickness of the fluorescent band is related to AFB_1_ concentration. Therefore, in the seven-day long assay, AFB_1_ concentration was reduced in all cases with respect to control. When plates were incubated over 21 days, a reduction in AFB_1_ concentration was observed in plates treated directly with *O. virens* EO as well as both EO niosome-encapsulated. However, no differences were found after *S. montana* EO application.

#### 2.2.2. Polypropylene Woven Bags Assays

**Figure 5:**
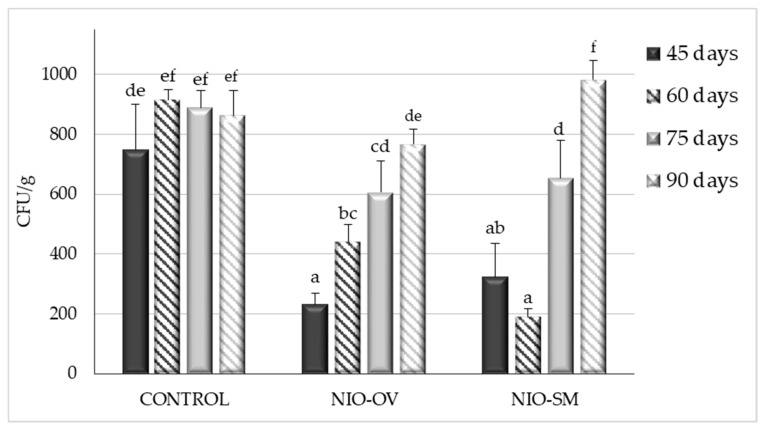
Effect of *S. montana* (NIO-SM) and *O. virens* (NIO-OV) EO encapsulated in niosomes on *A. flavus* growth in corn grains incubated for 45, 60, 75, and 90 days. Each value is the mean of three replications and the thin vertical bars represent the standard error of the corresponding data. Groups with the same letter are not significantly different (*p* > 0.05).

Figure 5 shows the results in CFU/g of *A. flavus* inoculated corn stored in polypropylene woven bags at all incubation times and after treatment with EOs encapsulated in niosomes.

After 45, 60, and 75 days of incubation, both of the EOs encapsulated in niosomes were able to control fungal growth, with a maximum reduction of up to 79% and 69% for *S. montana* and *O. virens* EOs, respectively. However, this effect seems to be lost over time and no significant differences with respect to the control group were found after 90 days of incubation.

**Figure 6:**
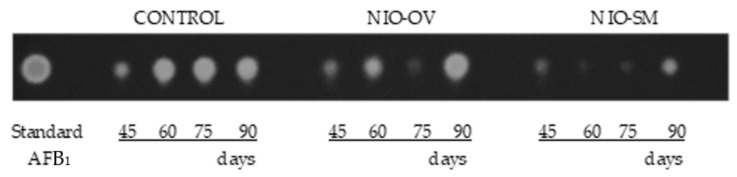
AFB_1_ detection by thin layer chromatography (TLC) in polypropylene woven bags inoculated with *A. flavus* after *S. montana* (NIO-SM) and *O. virens* (NIO-OV) niosome treatment of corn incubated for 45, 60, 75, and 90 days. The intensity and thickness of the fluorescent band are related to the concentration of toxin. The standard corresponds to the application of purified AFB_1_ (0.05 mg/mL).

The results regarding AFB_1_ detected on inoculated control bags or after niosome-encapsulated EO treatments are shown in Figure 6. The band intensity of each treatment was apparently lower with respect to their corresponding control in all cases except for NIO-OV after 90 days of incubation when a slight increase in production was detected. Treatment using niosome-encapsulated *S. montana* EO was the most effective to control AFB_1_ production by *A. flavus*.

Temperature and humidity were recorded during the experiment with ranges of 25–27 °C and 75–85%, respectively.

## 3. Discussion

Aflatoxin (AF) presence poses a high risk to food security and most countries have established maximum levels of these contaminants allowed in food products [3]. Appropriate control mechanisms are needed to keep these toxins from entering the food chain. In recent years, essential oils (EOs) have come to be considered as a safe, ecofriendly, renewable, and easily biodegradable option to be used as a food supplement [13]. Moreover, many EOs have been described as potent antifungal compounds that are able to interfere in mycotoxin synthesis [15,25,26,27]. In this work, we selected EOs extracted from *Rosmarinus officinalis*, *Thymus vulgaris*, *Satureja montana*, *Origanum virens*, *O. majoricum*, and *O. vulgare* to determine if they were able to control *Aspergillus flavus* growth and if they reduced AF production by this fungus. To some extent, all of the EOs tested modified the fungal growth rate and extended the lag phase at high concentrations. However, at lower doses, the EOs extracted from *S. montana* and *O. virens* were the most effective at reducing both fungal growth and AF production, and they were selected to perform subsequent studies. Chromatographic characterization of the EOs used in this study were carried out in the Agricultural Research Centre of Albaladejito (data not shown) and the results revealed that *S. montana* and *O. virens* EOs are highly rich in carvacrol and thymol, respectively. These compounds have been reported to be able to interact with the cell membrane, disrupt cell permeability, and produce cell death [28]. Pure extracts of both carvacrol and thymol have been reported to inhibit the growth of important mycotoxin-producing species such as *A. niger*, *A. flavus*, *A. ochraceus*, and *F. graminearum* [29,30].

Different authors consider that EO-based formulations could be safe, ecofriendly preservatives to avoid post-harvest losses due to mycotoxin contamination [14]. Therefore, taking into account the potent antifungal properties of EOs, many studies have focused on developing successful application protocols to minimize their drawbacks, which limit their direct use in food products [18] and, therefore, it is essential to protect them to extend their shelf life and activity [19]. The controlled liberation of EOs and their encapsulation in nanoparticles made of different materials are considered a good option [19,23]. These technologies attempt to reduce the rapid loss of their active principles. In general, EOs are a complex mix of lipophilic compounds and, therefore, lipid nanoparticle systems such as liposomes are the most appropriate [23,31]. In our study, the small-scale experiments showed that the effectiveness of EOs to control *A. flavus* growth diminished over time. However, when both EOs were encapsulated in niosomes, a significant reduction in fungal viable counts with respect to the untreated control group were found even after 21 days of incubation. Hence, it seems that the niosome-based nanoparticles were able to reduce the loss of the EOs’ active principles and produce a controlled release of their compounds. Similar results were obtained in the larger-scale experiments using maize stored in polypropylene woven bags. This type of storage is very common in African countries because it offers low-cost protection for grain from pests. However, a higher contamination by toxigenic fungi and mycotoxins in the grains has been reported owing to the change in moisture content, which increases the relative humidity inside the bags [32]. Even under the worst conditions for maize storage, both of the EOs encapsulated in niosomes were able to control fungal development, significantly reducing aflatoxin levels, and their effect was extended for up to 75 days. Hence, these promising approaches might be useful to prevent AF contamination under more appropriate storage conditions such as PICS (Purdue improved crop storage) bags or directly in silos.

The use of EOs in the agri-food industry is not a safety concern because several studies have ensured that they are safe as food additives and many of them are included in the GRAS list [14]. However, data are scarce regarding the effects that vesicle materials might have on human health and it is essential to carry out ecotoxicity studies to assess the impact of encapsulation matrices [19]. The niosome vesicles used in this work are commercially available and their non-toxic properties have been fully demonstrated. Moreover, they have been approved as a good option for the development of nanoparticles to improve medical therapies, including the controlled delivery of drugs or even vaccine antigens [24].

EO-based formulations need to overcome several tests before their application in food systems, as some active components can interact with food matrix components [14,33]. AFs often occur in maize, one of the most important basic cereal products worldwide for food and feeds [6]. In the present work, *S. montana* and *O. virens* EOs encapsulated in niosomes were directly applied to control *A. flavus* growth and its mycotoxigenic potential in artificially contaminated maize as a preliminary step to optimizing their application. EOs’ release was effective over time in both small-scale tests and simulated storage conditions and, therefore, no interactions with the components of the maize seemed to occur. It would be interesting to apply this newly developed technology to other food matrices often contaminated with AFs to confirm that this effect could be extrapolated to other products.

## 4. Conclusions

In this work, we proposed a novel niosome-based EO product that was successfully applied in polypropylene woven bags simulating common storage conditions of maize. The involvement of the company Nanovex Biotechnologies S.L. guarantees a correct and standardized encapsulation protocol and the reduction of problems that might arise during product formulation. The presence of encapsulated EOs in the bags significantly reduced *A. flavus* development and the effect was observed until 75 days after inoculation. The effect of this formulation could be easily maximized by applying the products regularly during maize storage, that is, every 45 days. Regularly scheduled application, together with good agricultural practices and the maintenance of adequate storage conditions, may be a sustainable way to avoid the occurrence of aflatoxins in stored maize.

## 5. Materials and Methods

### 5.1. Fungal Strains and Essential Oils

All *Aspergillus flavus* strains used in this study were able to produce aflatoxins from group B (AFB_1_ and AFB_2_) and G (AFG_1_ and AFG_2_) and were isolated from wheat from Morocco (S.44-1) and maize from Spain (A7). The correct identification of these isolates was confirmed using species-specific PCR protocols [34].

The strains were stored as a spore suspension at −80 °C in 15% glycerol (Panreac, Barcelona, Spain) until required. They were subcultured on potato dextrose agar (PDA, Pronadisa, Spain) and incubated at 28 °C for four days. The spore suspensions were prepared in sterile saline solution (9 g/L sodium chloride) (Merck, Darmstadt, Germany) supplemented by Tween 80 0.5% (Panreac, Spain). The spore concentration was determined using a Thoma counting chamber (Marienfeld, Lauda-Königshofen, Germany) and adjusted to a final concentration of 10^2^ or 10^6^ spores/mL depending on the assay.

The EOs tested were extracted from rosemary (*Rosmarinus officinalis* L.), thyme (*Thymus vulgaris* L.), savory (*Satureja montana* L.), and three species of oregano (*Origanum virens* Hoffmanns. & Link, *O. majoricum* Camb., and *O. vulgare* L.). The EOs were provided by The Agricultural Research Centre of Albaladejito (Cuenca, Spain). Each species was processed in batches of 100–150 g of plant aerial parts, following the methodology proposed by the European Pharmacopoeia by hydrodistillation, in a Clevenger-type apparatus for 2 h. These EOs were analyzed in gas chromatograph equipped with a flame ionization detector (FID) and capillary column VF-5 of 60 mm × 0.25 mm, 5% phenyl methyl siloxane. A temperature gradient of 70 to 240 °C was applied, with an increase of 3 °C per minute, maintaining the final temperature for 2 min. For the identification of the EO components, the relative retention times of standards and the corresponding Kovats indices were used. The quantification of the components was performed according to the areas of their chromatographic peaks.

These compounds were filtered (pore size 0.2 μm) (Fisherbrand, Shanghai, China) and stored at −20 °C in amber glass vials (Thermo Scientific, Madrid, Spain) until required.

### 5.2. Effectiveness of Plant Essential Oils on Fungal Growth and Aflatoxin Production

The effect of EOs at different concentrations on *A. flavus* S.44-1 growth and its ability to produce AFs were evaluated on CYA medium (45.5 g/L of modified Czapek–Dox agar (Pronadisa, Spain), 5 g/L of yeast extract (Pronadisa, Spain)). EOs were diluted in polyethylene glycol 400 (PEG (Acros, Geel, Belgium)) and added to the medium to obtain final concentrations of 10, 100, 500, and 1000 μg/mL. The same amount of PEG was included in the control plates instead of EO. CYA plates supplemented with EOs were inoculated with 1.5 μL (4 mm of diameter) of a 10^6^ spores/mL suspension on the center of the plate, and incubated at 28 °C for five days. All the conditions were tested in triplicate.

Fungal colony diameters were measured daily in two directions at right angles to each other until the medium was fully colonized (five days). Growth parameters were calculated from a linear model obtained by plotting the diameter (mm) against time (day). The parameters determined were λ, representing the lag phase (days prior to mycelial growth), and μ_max_, representing the maximum growth rate (mm/day), for control plates and each EO concentration tested.

AFs were extracted from the plates after six days of incubation, as described elsewhere [35]. Three agar plugs were removed from the centre, medium, and outer edge of the colony and toxins were extracted with 1 mL of methanol (Merck, Spain). Samples were stored at −20 °C until analysis. AFs were measured by high performance liquid chromatography (HPLC) using the protocol described below.

### 5.3. Effect of Satureja Montana and Origanum Virens Essential Oils Encapsulated in Niosomes on Fungal Growth and Aflatoxin Contamination

#### 5.3.1. Procedure for Microencapsulation of Essential Oils

EOs extracted from *S. montana* and *O. virens* were encapsulated in non-ionic surfactant-based lipid vesicles (niosomes). These particles were prepared by Nanovex Biotechnologies S.L. (Oviedo, Spain) starting from 40 mL of each type of EO. Niosomes were obtained using the thin film hydration (TFH) method with homogenization and sonication to obtain niosomes with a good polydipersity index (PDI) and a particle size between 100 and 200 µm, with an EO concentration of 12 µL/mL.

The characterization of the niosomes was performed with a Zetasizer Nano ZS particle size analyzer (Malvern Panalytical Ltd., Malvern, UK), which uses dynamic light scattering (DLS) to determine particle size, and the M3-PALS technique to calculate the ζ-potential.

A nanoparticle tracking analysis (NTA) was performed using a nano sight particle tracking analyzer (Malvern Panalytical Ltd., Malvern, UK) to determine concentration and size distribution.

#### 5.3.2. Effect of Niosome-Encapsulated Essential Oils on Fungal Growth and Aflatoxin Production on Maize Grains

Previously autoclaved maize grains were inoculated with *A. flavus,* strain A7. Then, 100 g of corn was immersed for 2 h in 100 mL of spore suspension 10^4^ spores/mL to obtain a final concentration of 10^2^ spores/g. Subsequently, the effect of niosome-encapsulated EOs was tested in a small-scale test in Petri dishes as well as in polypropylene woven bags simulating real storage conditions. At the beginning of the experiments, grain moisture was measured using a Hygropalm HP23A (Rotronic, Bassersdorf, Switzerland) and water activity was 0.95 in all cases.

##### Small-Scale Assays

Ninety millimeter Petri dishes were filled with crystalized potassium sulphate (Acros, Spain) to maintain *a_w_* at 0.97 [36]. A 50 mm Petri dish containing 7 g of inoculated maize was placed inside the larger one. *Satureja montana* and *O. virens* EOs were applied directly to the grains or encapsulated in niosomes at 500 µg/g. Control assays, mock-inoculated with water, were also included. Incubation was performed at 28 °C and the effect of niosome-encapsulated EOs or those directly applied on maize grains was evaluated at 7 and 21 days.

After the incubation period, a sample of 3.5 g was taken from each treatment and a viable count was performed using serial decimal dilutions and inoculation on Rose Bengal with Chloramphenicol medium. Plates were incubated in darkness at 28 °C for two days. The fungal growth of maize grain was expressed as colony forming units per gram of maize (CFU/g).

Afterwards, another sample of 3.5 g was taken from each treatment, and shaken for 20 min with 35 mL of chloroform for AF extraction. AFB_1_ was measured by thin layer chromatography (TLC), as described below.

##### Polypropylene Woven Bag Assays

One-hundred grams of inoculated maize was placed in small polypropylene woven bags. Subsequently, niosome-encapsulated EOs (*S. montana* and *O. virens*) were added at a dose of 500 μg/g and mixed. Bags were incubated at room temperature in darkness for 90 days in independent plastic boxes for each treatment (40 × 40 × 30 cm). Inoculated maize grains without EOs were used as control. Temperature and relative humidity were registered using a data logger El-USB-1 (Easylog; LASCAR electronic, Salisbury, UK) every 8 h until the end of the assay.

After the incubation period, the bags were cut open and the maize was diluted in 900 mL of sterile saline solution (9 g/L) containing 0.05% Tween 80. The mixes were incubated in an orbital shaker (140 rpm) at 4 °C for 60 min to release spores. Then, serial decimal dilutions and culture in Rose Bengal with Chloramphenicol were used to estimate fungal growth as CFU per gram of maize.

A sample of 14 g of corn was taken from each beaker, and shaken for 20 min with 35 mL of chloroform for AFB_1_ extraction and subsequent evaluation by TLC, as described below.

### 5.4. Detection of Mycotoxins

#### 5.4.1. Detection of Mycotoxins by High Performance Liquid Chromatography (HPLC)

After AFB_1_ extraction with methanol, mycotoxin measurements were performed in the “Laboratorio Arbitral Agroalimentario” (Madrid, Spain) following its standardized protocols. AF was measured by HPLC on a reverse phase C_18_ column (Inertsil ODS3; 5 μm, 4.6 mm × 250 mm; GL Sciences, Tokio, Japan) at 40 °C in a Waters chromatograph 515 HPLC coupled with a fluorescence detector 474 (Waters, Milford, MA, USA) at excitation and emission wavelengths of 362 and 435 nm, respectively. The mobile phase contained water, methanol, and acetonitrile (60:20:20), and the flow rate was 1 mL/min. AF was eluted and quantified by comparison with a calibration curve generated from AF standards (OEKANAL^®^, Sigma–Aldrich, Steinheim, Germany). The detection limit of the technique was 2.5 ng/g.

#### 5.4.2. Detection of Mycotoxins by Thin Layer Chromatography

After AFB_1_ extraction with chloroform, samples were filtered using 0.45 µm syringe filters (Fisherbrand, Spain) and an aliquot of 1 mL was evaporated in a vacuum concentrator, Eppendorf™ Concentrator Plus with Pump and GB Plug (Fisher Scientific, Madrid, Spain).

Silica gel 60 chromatography plates (Merck, Germany) were used, and AFB_1_ presence was determined according to the protocols described elsewhere [37,38].

Samples and AF standards were re-suspended with 500 µL toluene/acetonitrile (95:5) (Panreac, Spain). Then, 10 µL of each sample was spotted on the plate. Toluene/acetone/acetonitrile (1:1:1 (LabKem, Barcelona, Spain)) was used as a mobile phase. Toxins were visualized under ultraviolet light (Spectronics, Westbury, NY, USA).

### 5.5. Statistical Analysis

Statistical analysis was performed on the effect of EOs encapsulated in niosomes with StatsGraphics Centurion XVII V.17.2.04 program (Statpoint Technologies Inc., Warrenton, VA, USA). The Shapiro–Wilk and Levene tests were used to check normality and homoscedasticity, respectively. Data were analysed using analysis of variance (ANOVA).

When data did not meet normality and homoscedasticity criteria, a non-parametric Kruskall–Wallis test was performed. These analyses were performed using the software InfoStat/E 2011 (FCA, Córdoba, Argentina). This was necessary in the case of the fungal growth and aflatoxin production variables indicated in Section 5.2 of Material and Methods.

In all cases, the significance level was set at *p* < 0.05.

## Figures and Tables

**Table 1:** Aflatoxin (AF) concentration (B_1_, B_2_, G_1_, and G_2_) in Czapek Yeast Autolysate Agar (CYA) plates supplemented with different concentrations (0, 10, 100, 500, and 1000 µg/mL) of essential oils (EOs) (*R. officinalis, T. vulgaris, S. montana, O. virens, O. majoricum*, and *O. vulgare*). Each value is the mean of three replications ± standard error. Groups with the same letter are not significantly different (*p* > 0.05). ND: non detected.

EOs	μg/mL	AFB_1_ (µg/g agar)	AFB_2_ (µg/g agar)	AFG_1_ (µg/g agar)	AFG_2_ (µg/g agar)
*R. officinalis*	0	10.754 ± 0.925 ^c^	0.201 ± 0.021 ^c^	0.485 ± 0.055 ^c^	0.088 ± 0.014 ^b^
	10	5.205 ± 1.033 ^bc^	0.1 ± 0.022 ^bc^	0.213 ± 0.045 ^bc^	ND ^a^
	100	5.223 ± 0.171 ^abc^	0.11 ± 0.006 ^bc^	0.216 ± 0.012 ^abc^	ND ^a^
	500	1.09 ± 0.152 ^ab^	0.017 ± 0.002 ^ab^	0.058 ± 0.007 ^ab^	ND ^a^
	1000	ND ^a^	ND ^a^	ND ^a^	ND ^a^
*T. vulgaris*	0	10.754 ± 0.925 ^b^	0.201 ± 0.021 ^b^	0.485 ± 0.055 ^b^	0.088 ± 0.014 ^b^
	10	7.04 ± 0.977 ^b^	0.122 ± 0.019 ^ab^	0.256 ± 0.059 ^ab^	ND ^a^
	100	5.994 ± 0.554 ^ab^	0.117 ± 0.019 ^ab^	0.264 ± 0.069 ^ab^	ND ^a^
	500	ND ^a^	ND ^a^	ND ^a^	ND ^a^
	1000	ND ^a^	ND ^a^	ND ^a^	ND ^a^
*S. montana*	0	10.754 ± 0.925 ^b^	0.201 ± 0.021 ^b^	0.485 ± 0.055 ^b^	0.088 ± 0.014 ^b^
	10	8.27 ± 0.686 ^ab^	0.151 ± 0.011 ^ab^	0.314 ± 0.029 ^ab^	ND ^a^
	100	ND ^a^	ND ^a^	ND ^a^	ND ^a^
	500	ND ^a^	ND ^a^	ND ^a^	ND ^a^
	1000	ND ^a^	ND ^a^	ND ^a^	ND ^a^
*O. virens*	0	10.754 ± 0.925 ^b^	0.201 ± 0.021 ^bc^	0.485 ± 0.055 ^b^	0.088 ± 0.014 ^b^
	10	10.52 ± 1.334 ^b^	0.245 ± 0.039 ^c^	0.508 ± 0.065 ^b^	ND ^a^
	100	0.033 ± 0.044 ^ab^	0.003 ± 0 ^ab^	0.004 ± 0.002 ^ab^	ND ^a^
	500	ND ^a^	ND ^a^	ND ^a^	ND ^a^
	1000	ND ^a^	ND ^a^	ND a	ND ^a^
*O. majoricum*	0	10.754 ± 0.925 ^b^	0.201 ± 0.021 ^ab^	0.485 ± 0.055 ^b^	0.088 ± 0.014 ^b^
	10	10.939 ± 0.21 ^b^	0.234 ± 0.008 ^b^	0.611 ± 0.057 ^b^	ND ^a^
	100	7.999 ± 0.628 ^ab^	0.186 ± 0.022 ^ab^	0.375 ± 0.028 ^ab^	ND ^a^
	500	0.003 ± 0 ^a^	ND ^a^	ND ^a^	ND ^a^
	1000	0.008 ± 0.008 ^a^	ND ^a^	ND ^a^	ND ^a^
*O. vulgare*	0	10.754 ± 0.925 ^c^	0.201 ± 0.021 ^b^	0.485 ± 0.055^b^	0.088 ± 0.014 ^b^
	10	10.143 ± 0.86 ^bc^	0.192 ± 0.015 ^b^	0.475 ± 0.056 ^b^	ND ^a^
	100	7.867 ± 0.409 ^abc^	0.168 ± 0.015 ^ab^	0.466 ± 0.08	ND
	500	0.738 ± 0.08 ^ab^	0.012 ± 0.002 ^a^	0.043 ± 0.003	ND
	1000	0.298 ± 0.068 ^a^	0.004 ± 0.003 ^a^	0.014 ± 0.004	ND

**Table 2:** Characterization of *O. virens* and *S. montana* essential oil particles encapsulated in niosomes.

	ZETASICER			NANOSIGHT	
	PDI	Z-AVERAGE (nm)	POTENCIAL-ʐ (mV)	Size (nm)	CONCENTRATION (Particle/mL)
*O. virens*	0.251 ± 0.019	156.2 ± 3.9	−14.5 ± 0.5	142.4 ± 1.0	(2.96 ± 0.12) × 10^14^
*S. montana*	0.251 ± 0.011	153.3 ± 2.8	−14.6 ± 2.3	140.6 ± 3.8	(1.86 ± 0.07) × 10^14^

PDI: polydipersity index.
